# Correspondence

**Published:** 1912-12

**Authors:** Henry S. Wellcome

**Affiliations:** 54 Wigmore Street, London, W.


### CORRESPONDENCE.

---

54 Wigmore Street, London, W.

Editor Buffalo Medical Journal,

Dear Sir.—My attention has been called to a paragraph you have kindly inserted in your journal with reference to the Historical Medical Exhibition which is being organized to be held in London at the time of the Meeting of the International Medical Congress next summer.

I noticed that you have a collection of ancient books on medicine and other interesting objects, including Indian bones showing fractures, and syphilitic lesions. You have also by this time probably had further promises of exhibits and I should greatly appreciate your kindness if you could furnish me with a list and description of any such objects you might be able to send for the Exhibition.

Thanking you for the trouble you have taken and awaiting the favor of your reply, I am,

Yours very truly,

HENRY S. WELLCOME.

We trust that the profession will take an interest in this exhibit. It occurs to us that the local Academies might well devote an evening to an exhibit of old books, rare specimens and various objects of historic value. Even if it is not feasible to send an exhibit to London—and in no better way can Western New York bring itself before the profession of the world—such exhibits would have a considerable educational value. It might even be well to extend the invitation to other than members of the profession.

